# The Effect of Taurine on the Recovery from Eccentric Exercise-Induced Muscle Damage in Males

**DOI:** 10.3390/antiox6040079

**Published:** 2017-10-17

**Authors:** Yanita McLeay, Stephen Stannard, Matthew Barnes

**Affiliations:** School of Sport, Exercise, and Nutrition, Massey University, Palmerston North 4474, New Zealand; s.stannard@massey.ac.nz (S.S.); m.barnes@massey.ac.nz (M.B.)

**Keywords:** taurine, reactive oxygen species, eccentric exercise, performance recovery

## Abstract

Eccentric exercise is known to bring about microstructural damage to muscle, initiating an inflammatory cascade involving various reactive oxygen species. This, in turn, can significantly impair physical performance over subsequent days. Taurine, a powerful endogenous antioxidant, has previously been shown to have a beneficial effect on muscle damage markers and recovery when taken for a few days to several weeks prior to eccentric exercise. However, to date no studies have looked at the effects of supplementing over the days following eccentric exercise on performance recovery. Thus, this study aimed to determine whether supplementing with taurine over three days following eccentric exercise attenuated the rise in serum creatine kinase and improved performance recovery in males. In a blinded, randomized, crossover design, ten recreationally-fit male participants completed 60 eccentric contractions of the biceps brachii muscle at maximal effort. Following this, participants were supplemented with 0.1 g∙kg^−1^ body weight∙day^−1^ of either taurine or rice flour in capsules. Over the next three mornings participants underwent blood tests for the analysis of the muscle damage marker creatine kinase and carried out performance measures on the isokinetic dynamometer. They also continued to consume the capsules in the morning and evening. The entire protocol was repeated two weeks later on the alternate arm and supplement. Significant decreases were seen in all performance measures from pre- to 24-h post-eccentric exercise (*p* < 0.001) for both taurine and placebo, indicating the attainment of muscle damage. Significant treatment effects were observed only for peak eccentric torque (*p* < 0.05). No significant time × treatment effects were observed (all *p* > 0.05). Serum creatine kinase levels did not significantly differ over time for either treatments, nor between treatments (*p* > 0.05). These findings suggest that taurine supplementation taken twice daily for 72 h following eccentric exercise-induced muscle damage may help improve eccentric performance recovery of the biceps brachii.

## 1. Introduction

Eccentric actions, whereby muscle lengthens while under tension, can produce significant microstructural damage as a result of high force per fiber ratio. Not only can this directly reduce the ability of muscles to contract, it can also generate an inflammatory cascade, activating various ROS (reactive oxygen species) generating pathways including xanthine and nicotinamide adeneine dinucleotide phosphate (NADPH) oxidase, and phagocytic respiratory burst [1]. The resulting formation of ROS at high concentrations can surpass the ability of endogenous antioxidant defence, leading to oxidative stress. This, in turn, can elicit ROS-induced secondary tissue damage, including lipid peroxidation and protein oxidation, further reducing the ability of skeletal muscle to perform [2,3]. Dietary antioxidants have the ability to lower ROS within the human body, and a number of studies have shown attenuation of oxidative stress markers following supplementation [4,5,6,7]. Additionally, certain antioxidants, such as Vitamin E, appear to have membrane-protective effects, possibly reducing microstructural fibre damage [8]. As a result, antioxidant supplementation is common practice amongst athletes hoping that the reduction of ROS will reduce muscle tissue damage, diminishing any subsequent performance reductions. However, despite observed reductions in ROS following exercise with antioxidant supplementation, there appears to be no correlation to improved recovery and performance. Indeed, while few studies show dietary vitamin supplementation to have potential protective effects on muscle damage and subsequent force loss [9,10] the majority show little to no beneficial effect [11,12,13,14]. Some even suggest negative performance effects [15,16]. Furthermore, due to their non-specific ROS scavenging action, long-term use of antioxidants has been shown to potentially blunt important muscle adaptations [17,18,19,20]. This can have detrimental outcomes on training progression and, ultimately, performance, and several studies have observed this. In recreationally strength-trained men and women, ten weeks of high-dose vitamin C + E supplementation was shown to significantly blunt post-exercise increases in ROS-activated mitogen-activated-protein-kinase (MAPK) and lessen strength gains compared to a placebo group [21]. A similar blunting effect of vitamin C was seen in overload-induced rats [19], with significantly less muscle hypertrophy and lower MAPK than controls.

Taurine, a sulphonic acid, is produced endogenously from the amino acid cysteine. Containing a thiol group, it has high antioxidant activity and is found abundantly in skeletal muscle. Several studies have reported the cytoprotective actions of taurine against various tissue pathologies, including retinal [22], cardiac [23], hepatic [24], and skeletal [25]. Indeed, several studies have observed accelerated aging [26] and incidence of muscular disorders [27] in taurine-depleted skeletal muscle. However, to date few have looked directly at the potential of taurine to protect skeletal muscle against eccentric-exercise induced muscle damage and oxidative stress. A study in rats found one month of taurine supplementation significantly increased muscle taurine content and completely blocked post-eccentric exercise rises in the circulating lipid peroxidation marker TBARS (thiobarbituric acid substances) [28]. Similarly, in men, two weeks of taurine supplementation resulted in reduced plasma levels of the muscle damage marker creatine kinase, reduced muscle soreness, and increased strength levels following eccentric exercise [29]. Taurine supplementation did not affect post-exercise rises in inflammatory markers important for adaptive pathways, suggesting it to be a better alternative to common dietary antioxidants. In their study, Ra et al. [30] observed reduced delayed onset muscle soreness (DOMS) following eccentric exercise of the elbow flexors in men supplemented with taurine compared to their placebo counterparts. However, no significant difference in muscle damage markers were seen between treatments. While these studies have used taurine pre-supplementation, to date no research has looked solely at the effect of taurine supplementation following eccentric-exercise on performance recovery in humans. This study, therefore, aimed to determine the effects of post eccentric-exercise taurine supplementation on creatine kinase activity and muscle performance recovery in the elbow flexors (biceps brachii). 

## 2. Methods

### 2.1. Subjects

Ten healthy males (mean ± standard deviation (SD) age = 26.5 ± 6.5 years, height = 180 ± 9.2 cm, mass = 80 ± 11.5 kg) were recruited for this study. All participants were recreationally fit, engaging in exercise 2–3 times per week. Individuals were screened using a Health Screening Questionnaire designed to exclude those who could not take part due to physical, cultural, or religious reasons. Once deemed suitable, participants were asked to sign a consent form. Approval for this study was granted by the Massey University Human Ethics Committee, Southern A Application (approval number 16/33). 

### 2.2. Pre-Testing Procedures

At least one week prior to their first trial, participants underwent a familiarization session involving an outline of the study protocol, and familiarization with the performance measures (isometric, concentric, eccentric) on the isokinetic dynamometer (Biodex Medical Systems, Inc., New York, NY, USA). Height and weight were measured, and dynamometer seat settings were determined and recorded for subsequent use. Participants were advised to discontinue any supplements, energy drinks, and alcohol consumption during the week leading up to their first trial. Three days prior to the first trial, and for three days (72 h) following eccentric exercise-induced damage, participants were asked to record their food intake and exercise activity, and were asked to abstain from any eccentric-based exercise. The day before their first trial, they were asked to abstain from all exercise aside from necessary walking. Food records from the first trial were replicated during the second trial to control for nutritional intake, as was exercise. 

### 2.3. Overview

The study protocol was designed as a single-blinded, cross-over protocol, with the exercised arm and treatment randomly allocated. Participants attended the laboratory, underwent pre-exercise blood collection, and then proceeded to perform six sets of ten eccentric repetitions of the biceps muscles on an isokinetic dynamometer. This protocol has been previously used [31,32] to provoke significant levels of muscle damage observed by decreased muscular performance, and rises in circulating creatine kinase (CK) activity, a marker of muscle damage. At the completion of this protocol, participants were given either taurine or rice flour “placebo” capsules to ingest immediately, and again in the evening, along with a standardized breakfast (CHO 44 g, protein 8.4 g, Fat 16.5 g, 1450 kJ). Participants returned to the lab, fasted, the following three mornings (24 h, 48 h, 72 h) for follow up muscular performance measures of both the exercised and control arm and venous blood sampling. At the 24 h and 48 h visits, participants were given capsules to take that morning and in the evening. At least two weeks later, participants completed the same protocol on the other arm, and were given the other supplement to consume.

### 2.4. Blood Measures

On the morning of their first trial, participants attended the lab, fasted, where they underwent collection of a pre-exercise venous blood sample from the antecubital fossa into vacutainer tubes to determine serum levels of CK. The tubes were inverted several times, left at room temperature for 30 min, and then centrifuged at 4 °C for 10 min at 2000 rpm (revolutions per minute) (Eppendorf centrifuge 5804R, Eppendorf, New York, NY, USA). Serum was then aspirated into 1 mL aliquots and frozen at −80 °C for later analysis. Blood samples were also collected at 24 h, 48 h, and 72 h post-exercise. 

### 2.5. Muscular Performance

Following blood collection, participants carried out a two minute warm-up on an arm ergometer (Excalibur Sport, Lode, Groningen, The Netherlands). After completion of the warm-up, participants were seated on the isokinetic dynamometer (Biodex Medical Systems, New York, NY, USA) and straps were fixed across the chest to isolate the movement of the arm. Elbow joint range of motion was set and recorded for use in subsequent follow-up tests. Participants then performed separate sets of three maximal isometric (ISO), concentric (CON), and eccentric (ECC) contractions of the biceps muscles of both arms to complete the eccentric protocol (exercised) and the other (control) arm. ISO tension was measured at an elbow angle of 75°, while CON and ECC torque was measured at an angular velocity of 30°∙s^−1^ (degrees per second). Peak torque/tension and average peak torque/tension over the three contractions was recorded. Muscular performance was measured again at 24 h, 48 h, and 72 h post-exercise.

### 2.6. Exercise Protocol

Following the completion of pre-exercise performance measures involving three maximal contractions of ISO, CON, and ECC actions, participants completed six sets of 10 eccentric contractions over a 110° range of motion at an angular velocity of 30°∙s^−1^, using the biceps muscle of one arm. Each set was separated by two minutes of passive recovery. Participants were encouraged to resist the downward action of the dynamometer arm with as much effort as possible, and could visually see their torque output on a screen during the protocol to help maintain maximal effort. Total work over the exercise protocol was recorded. 

### 2.7. Treatment

Within 30 min of post-exercise blood collection, participants consumed either taurine powder (NOW foods) or rice-flour (Bob’s Red Mill, Milwaukie, OR, USA as a placebo in the form of vege-capsules. The amount of powder was set at 0.1 g∙kg^−1^ body weight∙day^−1^, based on several studies that found no adverse effects at levels of up to 10 g∙day^−1^ [33,34]. No participant was at a body weight that resulted in consuming more than 10 g∙day^−1^ (i.e., all were below 100 kg). Powder was split evenly into 1 g capsules that were then divided into two bags. Participants were advised to take one bag with breakfast and one with dinner. Identical capsules were taken over the following two days. During the second trial, the contralateral arm was exercised, and the other supplement was consumed. 

### 2.8. Creatine Kinase Analysis

Serum CK activity was determined by enzymatic method using reverse reaction. This in vitro assay was carried out using a Roche CK-NAC liquid assay kit (Roche Diagnostics GmbH, Manheim, Germany). 

### 2.9. Statistical Analysis

Data were analysed using the Statistical Program for Social Sciences (SPSS) for Windows (SPSS Statistics v.22, IBM, New York, NY, USA). A repeated-measures analysis of variance (ANOVA) was used to compare treatments over time for performance measures and CK. Statistical significance was set at *p* < 0.05 and the results are displayed as mean ± SD. Post-hoc analysis using the Holm-Bonferroni method was carried out on any significant results. 

## 3. Results

### 3.1. Performance Measures

Total work completed during the eccentric protocol did not significantly differ between trials, suggesting a similar level of effort during both. 

Completion of 60 eccentric muscular contractions of the biceps brachii resulted in significant decreases in ISO, CON, and ECC peak and average peak torque over time in the exercising arm only (all *p* < 0.01, Table 1). The greatest declines in force output for all performance measures were seen from pre- to 24 h in both the placebo (PLA) and taurine (TAU) groups (all *p* < 0.05). Only for peak eccentric performance was a significant treatment effect observed (*p* = 0.049), with a significantly greater force recovery with taurine at 48 h (*p* = 0.048). No treatment × time effects were observed for any performance measures (all *p* > 0.05).

### 3.2. Serum Creatine Kinase Activity

**Figure 1:**
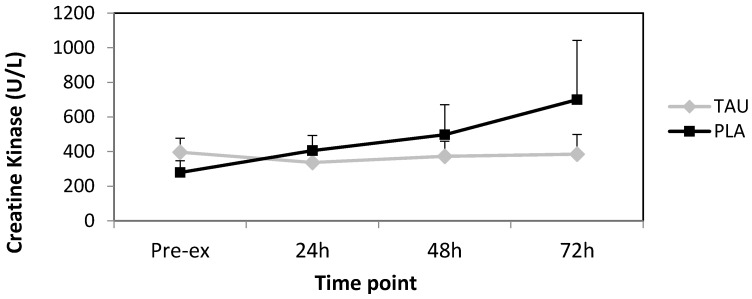
Mean absolute changes in serum creatine kinase (CK) activity following strenuous eccentric exercise with standard error bars. TAU = taurine, PLA = placebo.

Completion of 60 eccentric muscular contractions of the biceps brachii had no significant effect on serum CK activity from pre- to post-72 h (Figure 1). Differences between treatments were statistically non-significant (*p* = 0.352). Furthermore, no time or time × treatment effects were observed with *p* = 0.382 and *p* = 0.184, respectively. 

## 4. Discussion

Research has shown that supplementing with taurine for several weeks prior to eccentric-based activity prevents exercise-induced rises in oxidative stress and muscle damage markers in rats [28,35] and humans [30], and improves strength in young men [29]. Our study is the first that has looked solely at post-exercise taurine supplementation in humans, its effect on muscle damage markers, and subsequent performance recovery over 72 h. 

Significant declines in all performance measures in the exercised arm at 24 h post-exercise indicate the attainment of muscle damage. Treatment effects were only significant for peak eccentric torque, where force recovery significantly increased toward pre-values at 48 h with taurine compared to placebo. This suggests that taurine may expedite the recovery of eccentric force, potentially by mitigating inflammation-induced secondary damage. No such in vivo studies have looked at the effect of taurine supplementation on force recovery previously, however, Goodman et al. [36] observed that isolated skeletal muscle removed from rats following two weeks of taurine supplementation showed a greater recovery of force post high-frequency in vitro stimulation compared to controls. In a similar manner, Hamilton et al. [37] found force production in taurine depleted mouse skeletal muscle to be significantly lower than controls whilst Bakker and Berg [38] found taurine supplementation to increase force response in skinned rat skeletal muscle. It is clear that more in vivo studies need to be carried out to determine if in vitro results can be replicated. 

Research suggests eccentric-exercise induced performance reductions to peak between 24–72 h post-exercise, and return to full muscular force within seven days [39,40]. In line with this, our results show that neither treatment group fully recovered force output by 72 h. Perhaps measuring performance up to seven days post-damage may have shown potential differences in return to full force between treatments.

Serum CK activity is often used as a marker of exercise-induced skeletal muscle damage. Unlike several studies that have observed otherwise [28,29], we did not find any significant changes in CK over time or between treatments. It is possible that the use of a smaller muscle group gave a much lower relative release of CK into circulation compared to that of a larger muscle group worked to the same intensity may have. Furthermore, the significant difference between participant measures, gave rise to large standard errors, negating any possible difference between treatments. Indeed, a study by Nosaka and Clarkson [41] found a large inter-subject variability in CK response following eccentric exercise of the elbow flexors, largely due to the extent of muscular damage, which is dependent on a variety of factors. A larger number of participants or testing a larger muscle group may have reduced this error. 

It may have been useful to measure serum taurine throughout the study to determine whether the dose given was effective. Additionally, measuring markers of oxidative stress may have given an idea of any protective effect of taurine against exercise-induced ROS. Overall, this study suggests that taurine, when taken twice daily (at 0.1 g∙kg^−1^ body weight∙day^−1^) for 72 h following eccentric exercise may help accelerate eccentric performance recovery of the biceps brachii. Further human studies are needed to support this. As this study used males, it may be of interest to carry out a similar study on females. 

## 5. Conclusions

In the first of its kind, our study suggests that supplementation with taurine twice daily for 72 h following eccentric exercise-induced muscle damage may improve eccentric performance recovery of the biceps brachii in healthy males. We suggest this may be a result of taurine’s antioxidant and cytoprotective roles within skeletal muscle. The implications for taurine as a recovery supplement for athletes competing in eccentric-based sports is intriguing, but requires further research. 

## Figures and Tables

**Table 1:** Changes in torque (Nm) following strenuous eccentric exercise (mean ± SD). ISO = isometric, CON = concentric, ECC = Eccentric, PLA = placebo, TAU = taurine. * Significantly different to pre-exercise value (*p* < 0.01), # significantly different to pre-exercise value (*p* < 0.05). † Significantly different between trials (*p* < 0.05).

	Pre	24 h	48 h	72 h	Pre	24 h	48 h	72 h
Peak ISO								
*PLA*	74.1 ± 17	−21.3 ± 16.7 #	−16.3 ± 13.6 #	−10.9 ± 12.4	75.5 ± 19.5	−2.8 ± 4.9	−2.4 ± 6.7	−3.7 ± 7.4
*TAU*	75.4 ± 24.9	−18.2 ± 4.3 *	−18.5 ± 6.6 *	−9.7 ± 7.9 #	75 ± 17.1	−1.7 ± 8.3	−4.5 ± 6.6	−5.3 ± 7.1
Peak CON								
*PLA*	56.6 ± 16.2	−19.7 ± 8.4 *	−16.7 ± 11.4 *	−12.6 ± 7.9 *	56.5 ± 16	−3.7 ± 7.6	−4.7 ± 6.5	−4.9 ± 6.6
*TAU*	55.1 ± 17.9	−14.1 ± 3.9 *	−11.4 ± 7 *	−7.2 ± 9	57 ± 17.4	−5.1± 6.8	−5.6 ± 6.6	−6.5 ± 6.6
Peak ECC								
*PLA*	73.8 ± 16.5	−22.7 ± 13*	−25 ± 11.2 * †	−15.5 ± 9.6 *	72.5 ± 18.4	−0.4 ± 9.4	−0.7 ± 9.4	−4.9 ± 12.6
*TAU*	79 ± 24.3	−25.1 ± 14.9 *	−18.6 ± 12.3 * †	−18.4 ± 14.7 #	73 ± 21	−4.8 ± 6.4	−5.4 ± 6.8	−6.6 ± 6.8
Average ISO								
*PLA*	71.4 ± 16.4	−20.5 ± 14.6 *	−17.5 ± 13.6 #	−12.5 ± 12.5	72.5 ± 18.4	−2.4 ± 5.1	−1.7 ± 9	−3.9 ± 6
*TAU*	71 ± 20.7	−17 ± 7.7 *	−17.3 ± 6.6 *	−9.2 ± 7.3 #	70.7 ± 15.7	0 ± 8.1	−2.6 ± 7.5	−3.3 ± 6.5
Average CON								
*PLA*	53.5 ± 15.9	−19.1 ± 8.3 *	−16.9 ± 11.5 *	−11.8 ± 8.1 *	52.1 ± 14.4	−4.2 ± 6.3	−3.5 ± 5.3	−5.2 ± 5.4
*TAU*	51 ± 15	−12.9 ± 4.9 *	−10.8 ± 8.2 #	−4.6 ± 8.2	55.1 ± 14.6	−4.9 ± 5.8	−5.3 ± 6	−5.5 ± 6.6
Average ECC								
*PLA*	69 ± 16.2	−21 ± 14.5 *	−23.2 ± 11.6 *	−13.9 ± 9.2 *	67.3 ± 18.2	−0.6 ± 7.9	−0.6 ± 7.7	−3.3 ± 11.2
*TAU*	72.8 ± 23.2	−22.7 ± 12.7 *	−17.6 ± 12.6 *	−18 ± 14 #	70.1 ± 20	−4.7 ± 6.5	−6.3 ± 6.2	−7.5 ± 6.2 #

## Abbreviations

°C	Degrees Celsius
°∙S^−1^	Degrees per second
CK	Creatine kinase
DOMS	Delayed onset muscle soreness
g∙kg^−1^·day^−1^	Grams per kilogram, per day
MAPK	Mitogen activated protein kinase
PLA	Placebo
ROS	Reactive oxygen species
rpm	Revolutions per minute
SD	Standard deviation
TAU	Taurine
TBARS	Thiobarbituric acid substances
